# Post-Transplant Hepatic Steatosis: A Condition Not to Overlook

**DOI:** 10.3390/jcm12237340

**Published:** 2023-11-27

**Authors:** Iyiad Alabdul Razzak, Michael P. Curry, Michelle Lai, Hirsh D. Trivedi

**Affiliations:** 1 Department of Medicine, St. Elizabeth’s Medical Center, Tufts School of Medicine, Boston, MA 02111, USA; iyiad.alabdulrazzak@steward.org; 2 Liver Center, Beth Israel Deaconess Medical Center, Harvard Medical School, Boston, MA 02115, USA; 3 Karsh Division of Gastroenterology, Cedars-Sinai Medical Center, Los Angeles, CA 90048, USA; 4 Comprehensive Transplant Center, Cedars-Sinai Medical Center, Los Angeles, CA 90048, USA

## 1. Introduction

Recurrent or de novo steatotic liver disease (SLD) following liver transplantation (LT) is a rising concern among liver transplant recipients. Recent reports demonstrate a rising prevalence of post-LT steatosis and nonalcoholic steatohepatitis (NASH) [1,2,3]. It is estimated that 40% of LT recipients develop post-LT steatosis, which is comparable to the rate of SLD in the general population [3]. On the other hand, for patients who underwent LT for cirrhosis from NASH, post-LT steatosis was found in up to 100% of subjects within 5 years after LT [4]. The increasing prevalence of post-LT steatosis can negatively impact the survival of LT recipients and should not be overlooked. The advent of newly approved weight loss drugs also provides an avenue to manage SLD after transplant, if properly identified, to mitigate future metabolic and cardiovascular risks.

## 2. Assessing Risk

Chronic metabolic conditions often occur beyond the 1- or 3-year post-transplant period commonly observed among transplant centers. The development of metabolic risk factors, commonly linked to immunosuppressive regimens, may compromise graft function and survival in the long term. In a study of 226 LT recipients for NASH cirrhosis, half of the patients undergoing liver biopsy had recurrent NASH within an average follow-up of 3 years. About 10% developed bridging fibrosis, and 4 patients experienced recurrence of allograft cirrhosis over nine years following LT [5]. In a pooled analysis of 29 studies, the frequency of recurrent post-LT steatosis was 52%, while the de novo disease frequency was 31.6% [3]. More than one quarter of LT recipients will go on to develop post-LT NASH, and up to 23% can progress to advanced fibrosis [3,6,7]. In addition, those with recurrent NASH after transplant have more rapid fibrosis progression compared to NASH in the general population [8]. The association between post-LT steatosis and cirrhosis or graft failure may have been underpredicted due to low-quality evidence and limited follow-up periods [8]. It has been demonstrated, nonetheless, that post-LT steatosis is associated with higher cardiovascular event rates [9,10].

Understanding and evaluating risk factors for post-LT steatosis is critical. First off, the risk of post-LT steatosis varies based on the indication for LT. For instance, those transplanted for nonalcoholic fatty liver disease (NAFLD), alcohol-associated liver disease, and chronic hepatitis C have a higher risk of steatosis compared to those transplanted for hepatocellular carcinoma, autoimmune hepatitis, and cholestatic liver diseases [3]. Secondly, donor liver steatosis is a potential risk factor for post-LT steatosis. A pooled analysis of 559 steatotic grafts before transplantation showed a trend towards an increased risk of post-LT steatosis [3]. This association may become more pronounced with the rising prevalence of SLD in the general population. Graft steatosis may negatively impact short-term post-LT graft function. As such, multiple non-invasive imaging techniques have emerged as possible tools to screen for donor liver steatosis [11,12,13]. Information provided by these tools may help predict future risk of post-LT steatosis but requires further study.

Recipient risk factors are also major determinants of post-LT steatosis risk [3]. Not surprisingly, a higher recipient body mass index (BMI) and obesity are associated with post-LT steatosis. Metabolic derangements after transplant, including the development of metabolic syndrome, type 2 diabetes, arterial hypertension, and dyslipidemia (particularly hypertriglyceridemia), are significantly associated with post-LT steatosis [3,14]. Immunosuppressive medications potentially contribute to the development of post-LT steatosis by exacerbating insulin resistance and other metabolic risk factors [7,15]. Understanding patients’ risk factor profiles after LT is key to identifying NAFLD early and mitigating the risk of worse long-term outcomes.

## 3. A Tailored Approach to Diagnosis and Management

**Figure 1:**
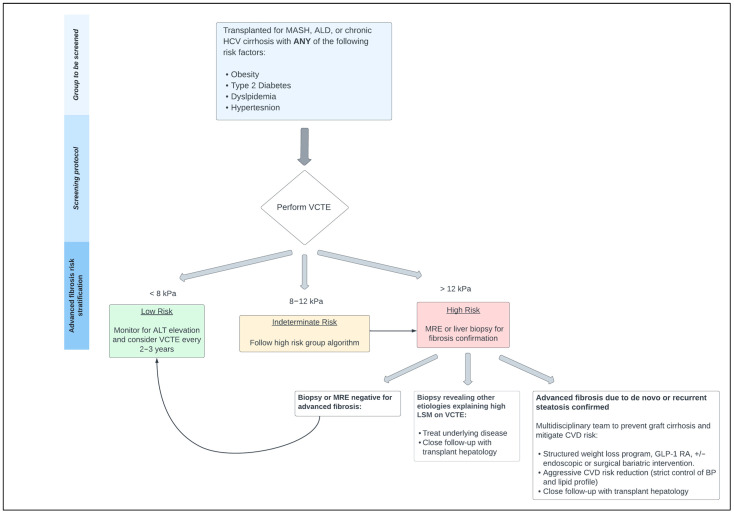
A proposed algorithm to screen for post-LT steatosis and advanced fibrosis involves starting with liver stiffness measurement (LSM) using transient elastography. Depending on LSM results, individuals are stratified into three risk groups. The low-risk group can be monitored every 2–3 years by repeating ALT measurements and/or VCTE. For the indeterminate and high-risk groups, we suggest obtaining MRE or liver biopsy for fibrosis confirmation. The figure also depicts a proposed management approach for patients with confirmed post-LT steatosis and advanced fibrosis. Abbreviations: VCTE = vibration-controlled transient elastography; LSM = liver stiffness measurement; MRE = magnetic resonance elastography; MASH = metabolic dysfunction-associated steatohepatitis; ALD = alcohol liver disease; HCV = hepatitis c virus; CVD = cardiovascular disease; GLP-1 RA = glucagon-like peptide 1 receptor agonists; ALT = alanine transaminase.

Studies on identifying SLD in the post-LT population are lacking. However, there is a growing body of literature examining the utility of non-invasive liver testing in post-LT settings. According to a metanalysis by Bhat et al., liver fibrosis post-LT was more accurately measured by transient elastography (TE) compared to serum biomarkers [6]. However, most included studies were evaluating liver fibrosis in the setting of recurrent HCV, and not SLD [6]. Magnetic resonance elastography (MRE) is another potentially valuable tool in assessing post-LT fibrosis. A recent study of 126 LT recipients found a statistically significant correlation between MRE and TE in measurements of graft fibrosis and steatosis [16]. Notably, the diagnostic performance of MRE and TE remained consistent across different etiologies of liver disease [16]. Yet, liver biopsy may ultimately be warranted to definitively diagnose SLD in high-risk patients or in those with discordant noninvasive testing results. To start off, incorporating non-invasive diagnostic tools offers several advantages, including reducing liver biopsies and enabling improved and early detection of post-LT steatosis. While awaiting more longitudinal data, we propose an algorithm to screen, evaluate, and manage post-LT steatosis and advanced fibrosis in Figure 1.

## 4. Mitigating the Risk

Treatments for metabolic conditions, such as obesity and type 2 diabetes, are rapidly evolving and may be considered in certain post-LT scenarios. Based on the International Liver Transplantation Society consensus, providers should follow the general population guidelines for managing metabolic comorbidities in patients with post-LT steatosis, until more data is available [17]. Weight loss drugs are commonly used in overweight or obese patients with NAFLD. Other than orlistat, no other oral weight loss agents have been studied in LT recipients [18]. Newer medications, like glucagon-like peptide 1 (GLP-1) agonists, have shown promising results and have led to significant weight loss, improvement in liver transaminases, and steatosis stage when studied in non-transplant patients with NAFLD but are not yet approved [19]. Currently, data on the utility of GLP-1 agonists in patients with post-LT steatosis are lacking, but this is subject to change as these medications undergo more widespread use. Surgical or endoscopic bariatric interventions can also be considered in carefully selected LT patients [18]. Finally, close follow-up with initiation or maintenance of guideline-based drugs for management of type 2 diabetes, hypertension, and dyslipidemia is essential.

Tailored strategies to reduce the risk of post-LT metabolic syndrome and hepatic steatosis from immunosuppressive medications are also important to optimize long-term patient and graft survival [14,18]. Early steroid tapering and minimizing the use of calcineurin inhibitors by using alternative agents can aid glycemic control in diabetic LT patients [14]. Charlton et al. found that combining everolimus with low-dose tacrolimus after LT resulted in modest weight reduction and the potential to mitigate insulin resistance development [20]. Nevertheless, based on available data, sirolimus appears to be the only immunosuppressant associated with an increased risk of post-LT steatosis [3].

## 5. Putting It All Together

In conclusion, post-LT steatosis is an often-overlooked condition with potential detrimental effects on long-term graft function and patient survival. Epidemiologic studies demonstrate a growing prevalence of NAFLD, now known as metabolic dysfunction-associated steatotic liver disease (MASLD), in the general population, but studies on post-LT patients are lacking. A call to action for this patient population who are at risk of MASLD, and its related outcomes, is warranted. To reduce the risk of post-LT steatosis, we recommend routine monitoring and aggressive modification of recipient cardiometabolic risk factors. We suggest the use of TE for early identification of post-LT steatosis in high-risk recipients, as outlined in Figure 1. To date, treatment strategies for post-LT steatosis are similar to those used in non-transplant settings. More prospective studies with longer-term follow-up are required to delineate the natural history of this condition, its true effect on patient and graft survival, and whether newly emerging weight loss medications would be effective in mitigating metabolic risks.
